# Known unknowns, Google Earth, plate tectonics and Mt Bellenden Ker: some thoughts on locality data

**DOI:** 10.3897/zookeys.247.4195

**Published:** 2012-11-30

**Authors:** Robert Mesibov

**Affiliations:** 1 Queen Victoria Museum and Art Gallery, Launceston, Tasmania, Australia 7250

**Keywords:** Spatial data, latitude/longitude, georeferencing

## Abstract

Latitude/longitude data in locality records should be published with spatial uncertainties, datum(s) used and indications of how the data were obtained. Google Earth can be used to locate sampling sites, but the underlying georegistration of the satellite image should be checked. The little-known relabelling of a set of landmarks on Mt Bellenden Ker, a scientifically important collecting locality in tropical north Queensland, Australia, is documented as an example of the importance of checking records not accompanied by appropriately accurate latitude/longitude data.

## Missing numbers

With the debut of *Biodiversity Data Journal* (http://www.pensoft.net/journals/bdj) not far off, I have started thinking, as a Pensoft editor, about the tables of locality data that are likely to be submitted. Will the authors have read the “Guide to Best Practices in Georeferencing"? (Chapman and Wieczorek 2006) produced for GBIF? Will the locality records be tabulated using Darwin Core categories for location (http://rs.tdwg.org/dwc/terms/index.htm#locationindex), or at least with fields that can easily be converted to their Darwin Core equivalents?

I suspect not. To judge from what I often see in recent research papers, a location will be specified simply as a latitude/longitude from a single reading of a handheld GPS receiver (standalone operation), together with an inexact description that checks the latitude/longitude by giving a general idea of the location, something like ca 5 km S of Woop Woop [an imaginary town], 22°48'20.6"S, 124°33'10.4"E

There are two numbers missing here. One is the datum for the latitude/longitude. While most GPS users set their receivers to the WGS84 datum, another datum may have been used in order to allow checking of the latitude/longitude or grid reference on a paper map based on a local datum. Datum differences are not trivial. For example, there is ca 200 m distance between the WGS84 location of a site in Australia and its corresponding location on a map referenced to the long-used AGD66 datum. Locality data tables should therefore include a field for specifying the datum or for noting that the datum is unknown. Compilers should not assume that if no datum is specified, then the datum for that location must have been WGS84.

The second missing number is an uncertainty estimate. We don't know whether the location is a spot or a large area, and if the latter, what relation the latitude/longitude has to the area sampled. We can be reasonably sure that the latitude/longitude is too accurate, even for a spot sample. The implied uncertainty is ±0.05", which at the given latitude represents about 1.5 m in either latitude or longitude. The GPS receiver's manufacturer probably didn't claim that high an accuracy. For example, the owner's manual for the Garmin GPSMap®62S, a popular handheld GPS receiver, says only that 95% of waypoints will be within 10 m of the stated position in typical use.

Good practice is to specify an uncertainty, and not just to imply one. A possible entry for an uncertainty field is the accuracy as reported by the GPS receiver for that particular reading; a column heading in the data table might be “GPS accuracy declaration" or something similar. In my part of the world, GPS accuracy declarations after a few minutes at a spot are generally in the range 5-10 m in the open air and 15–30 m in forest.

For many biological samplers, however, a collecting site is not a spot, but a small area over which a number of specimens are collected. A good way to report that location is to give the latitude/longitude of the centre of the site. The uncertainty can then be approximated as the radius of a circle containing the area searched, following the Darwin Core definition of uncertainty: “The horizontal distance (in meters) from the given decimalLatitude and decimalLongitude describing the smallest circle containing the whole of the Location" (http://rs.tdwg.org/dwc/terms/index.htm#coordinateUncertaintyInMeters).

ca 5 km S of Woop Woop, 22°48'20.6"S, 124°33'10.4"E, ±15 m, WGS84.

That's better, but something is still missing.

## Outrageous numbers

I recently audited some museum database records, one of which said the collecting site was at 22°06'57.54"S 117°53'15.31"E. At the latitude/longitude involved, those last 0.01" figures correspond to about 30 cm in latitude and longitude, or ±15 cm. How did the collector get those numbers?

Possibly from Google Earth. With coordinates set to degrees, minutes and seconds, the status bar at the bottom of a Google Earth window reports the cursor location to the nearest 0.01 second of latitude and longitude. You can zoom in as much as you like to see a collecting site, then just place the cursor on the site and read off the latitude/longitude, which will be outrageously accurate.

Since the latitude/longitude is not a GPS reading, it would be sensible to round the figures off to the nearest second. Uncertainty in this case depends in part on how accurately Google Earth has placed the satellite image on its mathematical model of the globe, a procedure known as georegistration. The accuracy of georegistration can vary from image to image and from date to date in an image series. One site I looked at in Queensland had shifted more than 100 m between image dates.

Google Earth can be very useful for locating sampling sites if GPS reception is poor or if GPS accuracy declarations are large. It's a good idea, however, to check the georegistration by getting at least one GPS reading at a spot (somewhere near the site) which will be clearly distinguishable on the satellite image. Even better, compare the known location of an official survey mark in the vicinity with its Google Earth location. In either case, the uncertainty specified for a position located using Google Earth should be at least the difference found between the Google Earth latitude/longitude and the corresponding figures for a GPS reading or a survey mark. (I recently checked a survey mark in my home town. Google Earth put it 2-3 m from its actual location. Not bad, but not as good as the implied ±15 cm.)

Here in Australia, at least, there is an additional complication. The Australian Plate is moving northeast towards Papua New Guinea at about 7 cm/yr and carrying with it the survey reference framework, GDA, standardised in 1994. Google Earth, like the GPS system, is based on the WGS84 framework, which for all practical purposes is independent of earth movements. In 2012, there is a more than a metre horizontal difference between GDA and WGS84, thanks to plate tectonics.

Complications aside, locality data tables should always contain a field for the source of latitude/longitude data, i.e. map, GPS, Google Earth, etc. The source will then stay with the record if the record is separated from the table's metadata.

ca 5 km S of Woop Woop, 22°48'20.6"S, 124°33'10.4"E, ±15 m, WGS84, GPS.

Much better!

## Numbers helped by words

A description like *ca 5 km S of Woop Woop* is obviously not as accurate as a GPS reading, but is important in a locality record as a check on latitude/longitude. The better the description, the closer the check. Description writing has been very well summarised by Chapman and Wieczorek (2006, p. 7):

– “Provide a descriptive locality, even if you have geographic coordinates. The locality should be as specific, succinct, unambiguous, complete, and as accurate as possible, leaving no room for uncertainty in interpretation."

– Localities used as reference points should be stable – i.e., places (towns, trig points, etc.) that will remain for a long time after the collection events. Do NOT use temporary locations or waypoints as the key reference location. You may have made an accurate GPS recording for the temporary location and then referenced future collections from that point (e.g., 200 m SE of the Land Rover), and that may make perfect sense for that series of collections. It is meaningless, however, when those collections are later broken up and placed in a museum under a taxonomic arrangement, and no longer have a link to where the ‘Landrover' was.

– If recording locations along a path (road, river, etc.) it is important to also record whether the distances were measured along the path (‘by road') or as a direct line from the origin (‘by air').

– Hint: The most specific localities are those described by a) a distance and heading along a path from a nearby and well-defined intersection, or b) two cardinal offset distances from a single persistent nearby feature of small extent. “[Example given for the latter: “ice field below Cerro El Plomo, 0.5 km S and 0.2 km W of summit, Region Metropolitana, Chile. "]"

Nevertheless, even the best landmark-based descriptions can stray from the truth if the landmarks themselves change, as the following example shows. I am describing it in some detail because the locality involved has yielded numerous new animal and plant species, and avoidable georeferencing errors are possible at this scientifically important locality.

## Unstable numbers

The Bellenden Ker Range in tropical north Queensland has attracted scientific explorers and collectors since the late 19th century. Much of the Range lies less than 10 km from the Coral Sea, and access is relatively easy from the coastal Bruce Highway between the towns of Gordonvale and Innisfail, and from the Atherton Tableland to the west. The densely vegetated Bellenden Ker Range has Queensland's highest mountain (Mt Bartle Frere, 1622 m) and Australia's wettest weather station (Mt Bellenden Ker, 8150 mm/yr).

**Figure 1.**
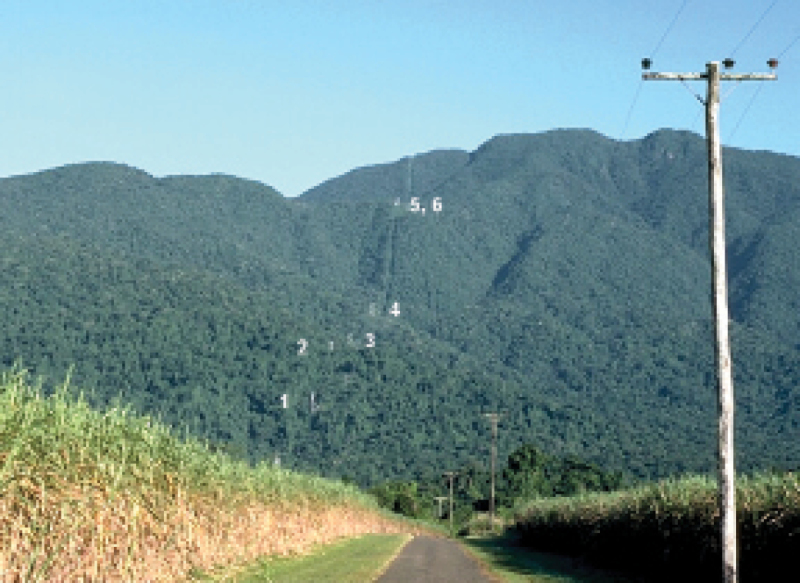
View of the Mt Bellenden Ker cableway from the east in June, 1976. Lower towers are labelled with their current numbers. Image by Len Webb, reproduced with the permission of the copyright holder, Griffith University.

In the early 1970s a cableway was built to access a telecommunications and broadcast facility on the summit of Mt Bellenden Ker (Fig. 1). Formerly operated by Telecom Australia (now Telstra), the Mt Bellenden Ker Cableway and Transmission Facility is currently owned and managed by Broadcast Australia.

Over the past four decades the cableway has not only offered access to Mt Bellenden Ker for biological sampling, but has also provided a simple system of landmarks, namely the nine cableway support towers. These landmarks have often been used to georeference collecting localities, with no accompanying latitude/longitude or only an approximate one. A botanical example is the type locality of *Morinda constipata* Halford & A.J. Ford, 2009 (Rubiaceae): ‘National Park Reserve 904, Wooroonooran, just S of tower 9, Mt Bellenden Ker cableway' (Halford and Ford 2009).

**Figure 2.**
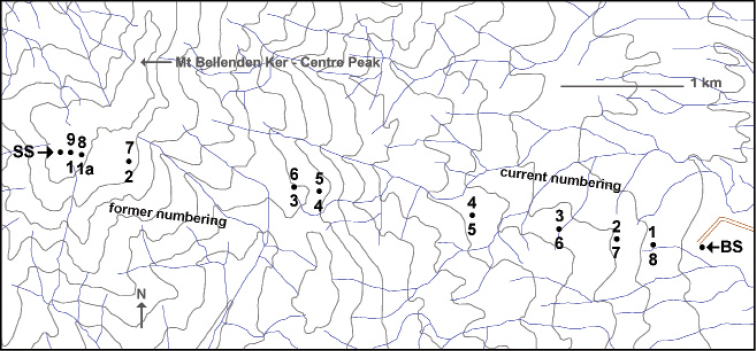
Plan of the Mt Bellenden Ker cableway showing current and former tower numbers. SS = summit station, BS = base station. Contours (100 m) and streamlines are only approximate and are from the 1:50000 scale ‘Bartle Frere' map produced by the Royal Australian Survey Corps in 1986.

It is not generally known, however, that the tower numbering was reversed in about 1997 when the cableway changed owners. Whereas Telecom numbered the towers from the top of the mountain to the bottom, the new system numbers the towers from bottom to top. Figure 2 shows the current and former tower numbering, and Table 1 gives latitude/longitude for each of the cableway landmarks.

Table 2 lists correct locations for the principal cableway sites sampled during the 1981 Earthwatch/Queensland Museum expedition, the source of a very large number of insect and other zoological samples (Queensland Museum 1982, Monteith and Davies 1992). The sites were referenced to the older tower numbering, and were offset various distances from the landmarks indicated on specimen labels.

**Table 1.** Spatial data (WGS84) for the landmarks mapped in Fig. 2. Positional uncertainty is ±50 m.

Landmark	Latitude, Longitude	Approx. elevation (m)
Cableway base station	17°16'12"S, 145°54'00"E	100
Tower 1 (formerly 8)	17°16'11"S, 145°53'46"E	210
Tower 2 (formerly 7)	17°16'10"S, 145°53'37"E	300
Tower 3 (formerly 6)	17°16'07"S, 145°53'22"E	360
Tower 4 (formerly 5)	17°16'04"S, 145°53'00"E	500
Tower 5 (formerly 4)	17°15'59"S, 145°52'20"E	970
Tower 6 (formerly 3)	17°15'58"S, 145°52'13"E	1030
Tower 7 (formerly 2)	17°15'53"S, 145°51'31"E	1450
Tower 8 (formerly 1a)	17°15'51"S, 145°51'18"E	1530
Tower 9 (formerly 1)	17°15'51"S, 145°51'15"E	1550
Summit TV station	17°15'50"S, 145°51'13"E	1550

**Table 2.** Spatial data (WGS84) for the principal cableway sampling sites of the 1981 Earthwatch/Queensland Museum expedition, based on the older tower numbering. Positional uncertainty is ±100 m.

Landmark	Latitude, Longitude	Approx. elevation (m)
‘Cableway base station'	17°16'06"S, 145°54'00"E	110 (‘100')
‘1 km S of towers 6/7'= ‘0.5 km S of tower 7 ‘ (now 2)= ‘1 km SW of tower 6' (now 3)	17°16'33"S, 145°53'15"E	500 (‘500')
‘Tower 3' (now 6)	17°16'02"S, 145°52'12"E	1020 (‘1054')
‘Summit TV station'	17°15'50"S, 145°51'14"E	1550 (‘1560')

## Conclusion

Locality databases have grown enormously in recent years, and the georeferencing of legacy localities from old specimen labels has become a well-understood practice for database compilers (Chapman and Wieczorek 2006). Many new localities are now being added to databases by collectors who have not been trained in georeferencing. There is more to capturing spatial data than pushing a button on a GPS receiver, and as an editor for the forthcoming *Biodiversity Data Journal* I hope to see locality records submitted with appropriate uncertainties, datum used, clear indications of data source and spatially explicit site descriptions.
